# Plasma zinc and selenium levels in adult inpatients with Lassa fever in Nigeria: A case-controlled study

**DOI:** 10.1016/j.ijregi.2024.100553

**Published:** 2024-12-20

**Authors:** Ebenezer Oseremen Dic-Ijiewere, Danny Asogun, Festus Oloruntoba Okojie, Adoghe Patricia Omono, Okpunu Eseoleleti Christopher, Adam Zumla, Rizwan Ahmed, Faith Unuabonah Huemomen, Joseph Okoeguale, Cyril Erameh, Ephraim Ogbainin, Sylvanus Okogbenin, Reuben Eifediyi, Linzy Elton, Isobella Honeyborne, John Tembo, Francine Ntoumi, Najmul Haider, Timothy D. McHugh, Alimuddin Zumla

**Affiliations:** 1 Department of Chemical Pathology, College of Medicine, Ambrose Alli University, Ekpoma, Nigeria; 2 Institute of Viral and Emergent Pathogens, Irrua Specialist Teaching Hospital, Ekpoma, Nigeria; 3 Department of Community medicine, Irrua Specialist Teaching Hospital, Irrua/ Faculty of Clinical Sciences, Ambrose Alli University, Ekpoma, Nigeria; 4 Department of Haematology, Irrua Specialist Teaching Hospital, Irrua/ Department of Haematology, Ambrose Alli University, Ekpoma, Nigeria; 5 Royal Bolton Hospital, Bolton NHS Foundation Trust, Bolton, United Kingdom; 6 Department of Haematology and Transfusion Science, Ambrose Alli University, Ekpoma, Nigeria; 7 Division of Infection and Immunity, Centre for Clinical Microbiology, University College London, London, United Kingdom; 8 HerpeZ and PANDORA-D-NET, UNZA-UCLMS Program, University Teaching Hospital, Lusaka, Zambia; 9 Fondation Congolaise Pour la Recherche Médicale, Brazzaville, Republic of Congo; 10 Institute for Tropical Medicine, University of Tübingen, Tübingen, Germany; 11 School of Life Sciences, Faculty of Natural Sciences, Keele University, Keele, UK; 12 National Institutes of Healthcare Research (NIHR), Biomedical Research Centre, UCL Hospitals NHS Foundation Trust, London, United Kingdom

**Keywords:** Lassa fever, Micronutrients, Inflammation, Zinc, Selenium, Biomarkers

## Abstract

**Objectives:**

Lassa fever (LF), a viral hemorrhagic disease endemic to West Africa, carries a high mortality. Scanty studies indicate that micronutrients may play a role in immunity to LF. We performed a case-controlled study to evaluate plasma zinc and selenium levels in hospitalized patients with LF.

**Methods:**

A cross-sectional controlled study of adults with confirmed LF and healthy controls presenting to the Institute of Lassa Fever Research and Control, Irrua Specialist Teaching Hospital, Nigeria, was performed between January and April 2023. After informed consent, blood samples were obtained, and LF was confirmed using a real-time polymerase chain reaction assay. Plasma zinc and selenium levels in plasma were measured using the AA500 Atomic Absorption Spectrophotometer. Data analysis was performed using SPSS version 21.0, comparing zinc, selenium, urea, and blood urea nitrogen levels of patients with LF and healthy control patients.

**Results:**

We enrolled 124 patients: 64 patients with LF and 60 healthy controls. There was no difference in mean age (37.6 years vs 35.2 years, *P* = 0.10) and gender (male 53% vs 56%, *P* = 0.82). Mean plasma zinc levels were significantly lower in patients with LF (0.97 mg/ml, SD = 0.12) compared with controls (1.85 mg/ml, SD = 0.13; *P* <0.0001). A consistent depletion of zinc levels was seen across the moderately severe cases (n = 28) and severe cases (36 cases) compared with healthy controls (0.97 ± 0.19 vs 0.96 ± 0.10, *P* = 0001). Plasma selenium levels were also significantly lower in patients with LF (mean: 76.80 ng/ml, SD: 6.66, % coefficient of variation: 8.67) compared with controls (mean: 93.10 ng/ml, SD: 12.46, % coefficient of variation: 13.38) (*P* = 0.008). Selenium levels in severe LF cases were 73.47 ± 14.68 and 80.98 ± 18.60 in moderately severe cases (*P* = 0.26).

**Conclusions:**

Significantly low levels of zinc and selenium were observed in patients with LF compared with healthy controls, suggesting a role in susceptibility and pathogenesis. Given that zinc and selenium are essential trace elements for optimal functioning of the immune system, they may play a role in improving management outcomes.

## Highlights

• Lassa fever presents a spectrum of clinical presentations with high mortality rates
• Micronutrient deficiency may be associated with poor management outcomes
• Low zinc and selenium levels in patients with Lassa fever correlate with disease severity
• Micronutrients as adjunct therapies during outbreaks require evaluation

## Introduction

Lassa fever (LF), caused by the Lassa virus (LASV) [1,2], has been placed on the World Health Organization Blueprint of pathogens [3], prioritizing the development of effective medical interventions for LF management and control [3]. Annually, an estimated 100,000 to 300,000 people are infected in West Africa, though this figure may be underreported [[1], [2], [3]]. Endemic to West Africa, Nigeria has reported 971 confirmed cases with 166 deaths (case fatality rate 17%) from January to October 2024, including over 30 cases among health care workers [[1], [2], [3], [4], [5]].

Patients with LF present with a spectrum of clinical manifestations, from mild flu-like symptoms to severe disease, including renal failure, encephalitis, and hemorrhage, which increase the risk of death in approximately 20% of cases [[1], [2], [3]]. Although there is a lack of reliable epidemiological data, it is likely that only a minority of infections result in severe disease. It has been estimated that as many as 80% of LASV infections are asymptomatic or mild. The reasons for the variation in disease severity are unknown but may be related to differences in LASV strain, route and dose of inoculation, host genetic susceptibility, or comorbid infections or conditions.

Both innate and adaptive immunity play important roles in response to LASV infection (both innate and adaptive immunity appear to play important roles in the pathogenesis of viral infections, but these require definition). In addition, there remain huge scientific and clinical knowledge gaps on LF and several research priorities have been defined recently [5,6]. Micronutrients such as zinc and selenium are essential trace elements for optimal functioning of the immune system and for modulating deleterious inflammatory responses to infection with virulent viral pathogens. Several studies from sub-Saharan Africa [7] have recorded low zinc and selenium levels, and these supplements are recommended for patients with a range of infectious diseases. Zinc and selenium deficiencies in viral infections, such as HIV, influenza, and COVID-19, and their potential roles as adjunct therapies have been well-documented [[7], [8], [9], [10], [11], [12]]. However, accurate data on their involvement in LF epidemiology and pathogenesis are sparse. Although individual case reports have reported micronutrient levels in patients with LF, there have been no reports of case-controlled studies assessing zinc and selenium levels in patients with LF.

Basic science and translational research on LF have been hampered primarily due to the dangerous nature of the LASV and its high transmissibility, requiring strict implementation of infection control procedures and a safe laboratory environment for handling blood samples. Thus, there have been scanty studies using blood samples during LF outbreaks. Our institution has now built laboratory capabilities to conduct studies on dangerous pathogens. Given the immune and oxidative stress pathways involved in LF, ascertaining baseline zinc and selenium levels and determining any association with the severity of the disease in patients with LF could provide initial insights for developing further studies. These include determining relationships of micronutrient deficiencies with clinical expression of LF disease, underlying pathophysiologic mechanisms, progression to severe disease, and the development of potential adjunct therapeutic strategies with micronutrient supplementation to improve management outcomes.

We performed a case-controlled study evaluating plasma zinc and selenium levels in adult hospitalized patients with LF at a hospital in Southern Nigeria.

## Methods

### Ethics approval: ethics statement

This study complied with the World Medical Association's Declaration of Helsinki. Ethical approval was obtained from the Research Ethics Committee of Irrua Specialist Teaching Hospital (ISTH), Irrua, Nigeria. Written consent was secured from participants, and data confidentiality was maintained. Permissions were also obtained from the Institute for Lassa Fever Research and Control (ILFRC) and the Bernhard Nocht Institute Research Laboratory.

### Study design, area, and population

This was a prospective cross-sectional study conducted at the ILFRC within the ISTH, located in Irrua, Edo State, Nigeria. The ILFRC is a leading center for LF research and treatment, serving as a referral center for patients with LF across Nigeria and West Africa.

The sample size was analyzed using the EPITOOLS site. The assumed odds ratio (OR) used was 4, the confidence interval (CI) was 0.95, and power was 0.95. The sample size obtained per group was 53, total for both LF-positive and healthy control group. To avoid attrition bias, the LF-positive sample size was increased to 64, representing 25% of the total Lassa-positive cases for the epidemiological year in the study area (254 confirmed cases in Edo State, Nigeria).

The study utilized archived plasma samples from patients who tested positive for LF at ISTH between December 2022 and April 2023. The severity of LF disease was classified based on urea levels; patients with plasma levels of urea >100 mg/dl were considered as having “severe disease.”

### Laboratory analysis

LASV infection was confirmed using the RealStar Lassa Virus real-time polymerase chain reaction kit 1.0 (Altona Diagnostics, Hamburg, Germany), a highly sensitive and specific reverse transcription-polymerase chain reaction method. Plasma zinc and selenium levels were quantified using the AA500 Atomic Absorption Spectrophotometer. Hollow cathode lamps specific to zinc and selenium emitted unique wavelengths, ensuring precise measurement.

### Statistical analysis

Data analysis was performed using SPSS version 21.0. Descriptive statistics was performed using analysis of variance, and independent Student's *t*-tests were used to compare zinc and selenium levels between groups, with significance set at *P* <0.05. Statistical analysis of plasma zinc, selenium, urea, and blood urea nitrogen levels in comparison with healthy control patients was performed. The normality of data distribution and outliers were assessed to ensure the robustness of the results.

## Results

We enrolled a total of 124 patients, comprising 64 patients with LF and 60 sex-matched healthy controls. There was no difference in mean age (37.6 years vs 35.2 years, *P* = 0.10) and gender (male 53% vs 56%, *P* = 0.82). Patients with LF had significantly lower plasma zinc levels (mean: 0.97 mg/ml, SD: 0.12, % coefficient of variation [CV]: 12.37) compared with healthy controls (mean: 1.85 mg/ml, SD: 0.13, % CV: 7.03) (*P* = 0.000). Plasma selenium levels were also significantly lower in patients with LF (mean: 76.80 ng/ml, SD: 6.66, % CV: 8.67) compared with controls (mean: 93.10 ng/ml, SD: 12.46, % CV: 13.38) (*P* = 0.008). Multiple logistic regression analysis showed low zinc levels (OR: 0.19, 95% CI: 0.07-0.43) and low selenium levels (OR: 0.94, 95% CI: 0.90-0.97) were significantly associated with LF after adjusting for age and gender.

Plasma levels of zinc, selenium, urea, and blood urea nitrogen (blood urea nitrogen) are shown in Table 1.

**Table 1:** Plasma zinc and selenium levels in patients with LF compared with healthy control patients.

Parameter	Healthy controls (Mean ± SD)	Healthy controls (% CV)	Patients with LF (Mean ± SD)	Patients with LF (% CV)	*P*-value
Zinc (mg/ml)	1.85 ± 0.13	7.03	0.97 ± 0.12	12.37	<0.001
Selenium (ng/ml)	93.1 ± 12.46	13.38	76.80 ± 6.66	8.67	0.008
Urea (mg/dl)	17.27 ± 2.13	12.33	120.49 ± 13.06	10.84	<0.001
Blood urea nitrogen (mg/dl)	8.05 ± 0.18	2.24	56.21 ± 6.09	10.83	<0.001

Key: Values are significant at *P* <0.05.

CV, coefficient of variation; LF, Lassa fever.

Zinc levels: Mean plasma zinc levels were significantly lower in patients with LF (0.97 mg/ml, SD: 0.12) compared with controls (1.85 mg/ml, SD: 0.13; *P* <0.0001). A consistent depletion of zinc levels was seen across the moderately severe cases (n = 28) and severe cases (36 cases) compared with healthy controls (0.97 ± 0.19 vs 0.96 ± 0.10; *P* = 0001).

Selenium levels: Plasma selenium levels were also significantly lower in patients with LF (mean: 76.80 ng/ml, SD: 6.66, % CV: 8.67) compared with controls (mean: 93.10 ng/ml, SD: 12.46, % CV: 13.38) (*P* = 0.008). Selenium levels in severe LF cases were 73.47 ± 14.68 and 80.98 ± 18.60 in moderately severe cases (*P* = 0.26). This suggests a role for these micronutrients in the broader pathophysiology of LF rather than in differentiating stages of disease severity.

## Discussion

There are several important points arising from our study which require further discussion.

First, this is the first case-controlled study of zinc and selenium levels in hospitalized patients with LF. This study observed a significant reduction in plasma zinc and selenium levels among hospitalized patients with LF compared with healthy controls. This significant reduction in plasma zinc and selenium levels among patients with LF could also be attributed to increased metabolic demand and renal dysfunction, a pattern also noted in other infections like HIV and COVID-19 [[7], [8], [9], [10], [11]].

Second, the decreased levels of zinc could impair immune cell function, weakening the host's antiviral response. Concurrently, reduced selenium levels may hinder antioxidant defenses, leading to elevated reactive oxygen species levels that contribute to tissue damage and worsen the inflammatory response. Zinc's role in T-cell function and cytokine regulation is critical during viral infections, whereas selenium's antioxidant properties could suppress viral replication and reduce oxidative damage. Therefore, depletion of these micronutrients in LF could exacerbate the severity of the disease, leading to worse clinical outcomes.

Third, these micronutrient deficiencies are known to play a role in immune dysregulation [[7], [8], [9], [10], [11], [12], [13], [14]], potentially exacerbating the cytokine storm leading to multi-organ failure in several viral zoonotic diseases. In the context of LF, the significantly reduced levels of zinc and selenium observed may have contributed to the immune system's inability to mount an effective response against the LASV, contributing to increased disease severity.

Fourth, previous reports have shown that zinc and/or selenium supplementation in individuals with other viral infectious diseases, such as HIV, hepatitis C, and COVID-19, could modulate immune responses and improve outcomes [[7], [8], [9], [10], [11], [12], [13], [14]]. Thus suggesting a similar potential role for zinc and selenium in the management of LF.

Fifth, our results should be viewed in a public health context in West Africa, where LF is endemic, and countries are characterized by a high prevalence of micronutrient deficiencies, including zinc and selenium, largely due to poor dietary diversity, food insecurity, and economic challenges. Many diets in these regions rely heavily on phytate-rich grains, which inhibit the absorption of zinc and other essential minerals, predisposing populations to deficiencies even before they encounter infections like LF. These pre-existing nutritional deficits may impair the immune system's ability to respond effectively to LASV, thus heightening susceptibility to infection and severe disease.

Sixth, treatment options are limited [2,3], with Ribavirin, which appears to be an effective antiviral when administered early [3]. Given the limited access to timely antiviral treatments like Ribavirin for patients with LF in these regions, addressing micronutrient deficiencies through dietary interventions or supplementation programs represents a potentially low-cost strategy for enhancing immune resilience against LF. By integrating zinc and selenium supplementation into management protocols for LF, health care systems could adopt a dual approach—targeting nutritional deficiencies while reducing the severity of the disease.

Our study had several limitations, including a relatively small sample size and its single-center design, which limits the generalizability of the findings to the broader population in LF-endemic regions. Regional differences in diet, genetics, and access to care may have influenced the results. In addition, the study did not have data on dietary intake or pre-existing comorbidities and other confounders like dietary habits and taking nutritional supplements, which may influence micronutrient levels. These data were not available because these patients were acutely ill, and the focus was on their immediate medical problems.

Future studies should aim to address these limitations. Larger numbers of patients with LF and healthy controls across diverse populations and regions need to be studied, and to makeup these required numbers, we suggest collaborative studies across LF treatment centers in Nigeria or involve other centers in LF-endemic West African countries such as Sierra Leone and Liberia, using same study protocols. Randomized controlled trials could be particularly valuable in assessing the efficacy of zinc and selenium supplementation as adjunct therapies alongside Ribavirin in patients with LF. These could allow for mechanistic studies of how micronutrients could modulate pathogenesis. In addition, research should focus on understanding the dynamics of micronutrient levels throughout the course of infection and recovery, which could inform more precise nutritional interventions. Our findings suggest that zinc and selenium supplementation could serve as an adjunctive therapy to improve the immune response and reduce the severity of LF. Given the endemic nature of LF in regions with widespread micronutrient deficiencies, nutritional interventions could play a vital role in supporting immune function and improving patient outcomes, offering a valuable complement to existing antiviral treatments by strengthening the host's immune defenses. However, supplementation should not replace antiviral treatments but rather complement them by strengthening the host's immune defenses.

## Conclusion

In conclusion, our case-controlled study showed significant zinc and selenium depletion in hospitalized patients with LF, highlighting the potential role of these micronutrients in disease pathogenesis. Given that zinc and selenium are essential trace elements for optimal functioning of the immune system, they may play a role in improving management outcomes. These findings underscore that larger studies are required to confirm our findings. Rigorous controlled clinical trials are required to determine the efficacy, optimal dosing, and timing for micronutrient supplementation to establish their true potential in reducing LF infection risk, disease severity, morbidity, and mortality. Addressing underlying nutritional deficiencies alongside antiviral therapies could offer a more comprehensive approach to managing LF in West Africa. At a broader level, public health strategies should incorporate community-based programs that address food security and promote the consumption of locally available nutrient-rich foods. Such programs could enhance the population's overall nutritional status, protect against viral infections like LF, and potentially mitigate the disease burden and impact of future outbreaks.

## Funding

EU-EDCTP2–EU Horizon 2020 Framework Programme, PANDORA-ID-NET grant.

## Author contributions

Each author made substantial contributions to PANDORA-ID-NET and its studies on viral pathogens in West Africa, including the study concept, design, conduct, data analysis, interpretation, development and review of the figure, and writing of several drafts of the manuscript. All authors have seen the final draft and approved the submission. They have agreed both to be personally accountable for the author's own contributions and to ensure that questions related to the accuracy or integrity of any part of the work, even ones in which the author was not personally involved, are appropriately investigated, resolved, and the resolution documented in the literature.

## Data availability

The datasets used and/or analyzed during the current study are available from the corresponding author on reasonable request.

## Statement

During the preparation of this work the author(s) did not use AI or other writing tools.

## Declarations of competing interest

The authors have no competing interests to declare.
